# Repurposing existing drugs: identification of SARS-CoV-2 3C-like protease inhibitors

**DOI:** 10.1080/14756366.2020.1850710

**Published:** 2021-01-12

**Authors:** Wei-Chung Chiou, Meng-Shiuan Hsu, Yun-Ti Chen, Jinn-Moon Yang, Yeou-Guang Tsay, Hsiu-Chen Huang, Cheng Huang

**Affiliations:** a Department of Biotechnology and Laboratory Science in Medicine, National Yang-Ming University, Taipei, Taiwan; b Departments of Infectious Disease, Far Eastern Memorial Hospital, Taipei, Taiwan; c Institute of Bioinformatics and Systems Biology, National Chiao Tung University, Hsinchu, Taiwan; d Department of Biological Science and Technology, College of Biological Science and Technology, National Chiao Tung University, Hsinchu, Taiwan; e Center for Intelligent Drug Systems and Smart Bio-devices, National Chiao Tung University, Hsinchu, Taiwan; f Faculty of Internal Medicine, College of Medicine, Kaohsiung Medical University, Kaohsiung City, Taiwan; g Hepatobiliary Division, Department of Internal Medicine, Kaohsiung Medical University Hospital, Kaohsiung Medical University, Kaohsiung City, Taiwan; h Institute of Biochemistry and Molecular Biology, National Yang-Ming University, Taipei, Taiwan; i Department of Applied Science, National Tsing Hua University South Campus, Hsinchu, Taiwan

**Keywords:** SARS-CoV-2 3CL protease, antiviral, repurposing drugs, FRET, 3CLpro inhibitors

## Abstract

Severe acute respiratory syndrome coronavirus 2 (SARS-CoV-2) is responsible for coronavirus disease 2019 (COVID-19). Since its emergence, the COVID-19 pandemic has not only distressed medical services but also caused economic upheavals, marking urgent the need for effective therapeutics. The experience of combating SARS-CoV and MERS-CoV has shown that inhibiting the 3-chymotrypsin-like protease (3CLpro) blocks the replication of the virus. Given the well-studied properties of FDA-approved drugs, identification of SARS-CoV-2 3CLpro inhibitors in an FDA-approved drug library would be of great therapeutic value. Here, we screened a library consisting of 774 FDA-approved drugs for potent SARS-CoV-2 3CLpro inhibitors, using an intramolecularly quenched fluorescence (IQF) peptide substrate. Ethacrynic acid, naproxen, allopurinol, butenafine hydrochloride, raloxifene hydrochloride, tranylcypromine hydrochloride, and saquinavir mesylate have been found to block the proteolytic activity of SARS-CoV-2 3CLpro. The inhibitory activity of these repurposing drugs against SARS-CoV-2 3CLpro highlights their therapeutic potential for treating COVID-19 and other Betacoronavirus infections.

## Introduction

Coronavirus disease 2019 (COVID-19), resulting from severe acute respiratory syndrome coronavirus 2 (SARS-CoV-2) infection, has distressed medical services and economies worldwide and has had profound psychological effects since its emergence^1^^,^^2^. Among COVID-19 patients, about 81% have no or mild symptoms, with severe symptoms in 14% and critical illness in 5%^2^. The clinical manifestations of SARS-CoV-2 infection often include, but are not limited to, fever, cough, fatigue, muscle soreness and abdominal pain, similar to severe acute respiratory syndrome coronavirus (SARS-CoV) and Middle East respiratory syndrome coronavirus (MERS-CoV)^2^. Risk factors for becoming critically ill with COVID-19 include cardiovascular disease, diabetes and obesity; however, healthy people of any age can become critically ill with COVID-19, although the current data suggest that individuals over 65 years of age, particularly men, are more likely to have severe symptoms^3^. Because SARS-CoV-2 infection has become a global pandemic, causing severe damage to public health^4^, there is a desperate need for effective therapeutics.

SARS-CoV-2, an enveloped, positive-sense, single-stranded RNA (+ssRNA) Betacoronavirus (β CoVs), is quite similar to SARS-CoV^2^^,^^5^^,^^6^. The genome of SARS-CoV-2 is about 30 kb, in which open reading frames (ORF) 1a and 1 b encode two polyproteins (pps), pp1a and pp1ab^2^. To complete the lifecycle of SARS-CoV-2, successful proteolytic processing of pp1a and pp1ab is required to yield a total of 16 non-structural proteins (nsp1–16)^2^. The consensus functions of these virus-encoded proteolytic proteins are found in all β CoVs, specifically papain-like protease (PLpro) and chymotrypsin-like protease (3CLpro)^2^. In particular, the substrate binding site of SARS-CoV-2 3CLpro is highly conserved across the β CoVs suggesting the therapeutic potential of 3CLpro inhibitors for SARS-CoV-2 and other β CoVs^2^^,^^7^. In addition, alignment of the genomic sequences of SARS-CoV-2, SARS-CoV and MERS-CoV reveals a high-level conservation of the proteolytic sites and proteolytic enzymes^2^^,^^8^^,^^9^.

A member of the cysteine protease family, the active SARS-CoV-2 3CLpro comprises two identical monomers, each with three structural domains; the first two domains (domain I: 8–101 and II: 102–184) form a chymotrypsin fold, and the third (domain III: 201–303) forms a globular α-helical structure, with an identity of 96% to SARS-CoV 3CLpro^7^^,^^10^. In particular, the catalytic dyad of SARS-CoV-2 3CLpro includes H41 and C145 in domains I and II, respectively^7^; meanwhile, dimerisation and formation of the S1 subsite of the substrate binding site involve the interaction between the N-terminal residue (N-finger) of one polypeptide and the E166 residue of the other^11^. Consistently, the most variable regions of 3CLpro in known β CoVs were found to be situated in domain III and the surface loops, indicating that the proteolytic activity is mainly governed by domains I and II^7^.

Inhibition of the activity of 3CLpro in SARS-CoV-2 is regarded as a plausible approach to block its replication. Screening of FDA-approved drugs for SARS-CoV-2 3CLpro inhibitors has been conducted *in silico* and *in vitro*^7^, identifying two FDA-approved drugs (disulfiram and carmofur), and five preclinical or investigational compounds as promising antiviral agents against 3CLpro. In this study, we screened a library consisting of 774 FDA-approved drugs for potential SARS-CoV-2 3CLpro inhibitors. To evaluate the extent of inhibition of SARS-CoV-2 3CLpro, a fluorogenic peptide with intramolecularly quenched fluorescence (IQF) was used as the substrate for the protease. Subject to the inhibitory effect, the half maximal inhibitory concentrations of the repurposing existing drugs of interest were characterised, along with analysis of docking poses in the substrate binding site of SARS-CoV-2 3CLpro.

## Materials and methods

### Drug library

The SCREEN-WELL® FDA v. 2.0 Approved Drug Library (BML-2843–0100) was purchased from ENZO Life Sciences Inc., NY, USA, and comprises 774 clinical drugs with well-studied bioactivity, safety and bioavailability.

### Construction of pET28b(+)-SARS-CoV-2-3CLpro

A published sequence of SARS-CoV-2 3CLpro^11^ was chemically synthesised and cloned into an yT&A plasmid by Genomics, Taiwan. The insert, encoding full length SARS-CoV-2 3CLpro, was amplified from the yT&A plasmid using ExcelTaq™ Taq DNA polymerase (Smobio, Taiwan), primer 5′-ATGGGTCGGGATCCCAGTGGTTTTAGAAA-3′ and primer 5′-GGTGCTCGAGTTCATCTAGTTATTGGAAAGTAACACCTGAG-3′ and cloned into a T7-based pET-28b(+) plasmid (Thermo Fisher Scientific, MA, USA) digested with BamHI and XhoI (New England Biolabs, MA, USA). Plasmid extraction from *E. coli* DH5α cells was carried out using Presto™ Mini Plasmid kits or Geneaid™ Midi Plasmid kits. The amplicon was purified using a PCR clean-up DNA/RNA extraction kit (Viogene, Taiwan). The insert sequence of the pET28b(+) DNA plasmid was verified by the National Yang-Ming University Genome Research Center, Taiwan.

### Protein expression and purification

The SARS-CoV-2 3CLpro was purified using the His-tag at its N-terminal, using a nickel column from GE healthcare, IL, USA, following the procedure described previously^12^. The purified protein was resolved by SDS-PAGE and the image quantification with Multi Gauge densitometry (Fujifilm, Japan) characterised the protein purity to be over 95%. Biochemical protein quantification was performed using Bio-Rad protein assays (CA, USA), with the measurements at 595 nm in a SPARK® multimode microplate reader (TECAN, Switzerland).

### Protease activity assays using IQF peptide substrates

An Edans-Dabcyl FRET platform was established, following a published protocol^13^. Briefly, a consensus cleavage sequence recognised by SARS-CoV-2 3CLpro was synthesised by Genomics, Taiwan, with Dabcyl at the N-terminus and Edans at the C-terminus, Dabcyl-TSAVLQ↓SGFRKME-Edans. In protease activity assays, 0.25 µM protease was incubated with 1.25 µM peptide substrate for three hours. Assays were conducted in triplicate in Eppendorf® black 96-well microplates (MA, USA) using an assay buffer containing 12 mM Tris-HCl (pH 7.5), 120 mM NaCl, 0.1 mM EDTA and 1 mM dithiothreitol (DTT), in a final volume of 100 µL. The fluorescence signal at 538 nm, at a bandwidth of 15 nm, emitted from the cleaved IQF peptide substrate after excitation at 355 nm, at a bandwidth of 10 nm, was recorded by a SPARK® multimode microplate reader (TECAN, Switzerland). The relative fluorescence units (RFU) at a gain of 131 were calculated using Spark® Control Magellan™ v2.2 software.

### Dose-response curve analysis

SARS-CoV-2 3CLpro was incubated with drugs at 0–100 µM for an hour at 37 °C. Then, 1.25 µM IQF peptide substrate was added to the mixture to a final volume of 100 µL and incubated at 37 °C for another three hours, prior to detection. With the same parameters applied in protease activity assays, the RFU readouts obtained from the SPARK® multimode microplate reader (TECAN, Switzerland) were normalised to the negative control (vehicle only) in each assay plate. After drug treatment at a concentration between 0–100 µM, points of relative protease activity were fitted to a normalised dose-response model in GraphPad Prism 7.03 for IC_50_ characterisation, where $Y=\mathrm{Bottom}+\frac{\mathrm{Top}-\mathrm{Bottom}}{1+10^{(\mathrm{LogIC}50-X)\cdot \mathrm{HillSlope}}}$ .

### Molecular docking

For molecular docking, the interaction profile of a compound in the substrate binding site of SARS-CoV-2 3CLpro was simulated in GEMDOCK: molecular docking tool^14^. Retrieving the crystal structure of SARS-CoV-2 main protease from the Protein Data Bank (PDB ID: 6LU7^7^), the substrate binding site of SARS-CoV-2 3CLpro was defined by an 8 Å-radius sphere around the bound peptide-like inhibitor PRD_002214. The 3D drug structures (SDF files) from DrugBank^15^ were converted to MOL files by Open Babel^16^.

### Statistical analysis

Data collected in the study were analysed and plotted with GraphPad Prism 7.03 (GraphPad software) when a minimum of *N* = 3 independent samples was obtained. Values were expressed as the mean ± standard mean error (SEM) if not otherwise specified.

## Results

### Screening a 774 FDA-approved drug library against 3CLpro activity

**Figure 1.**
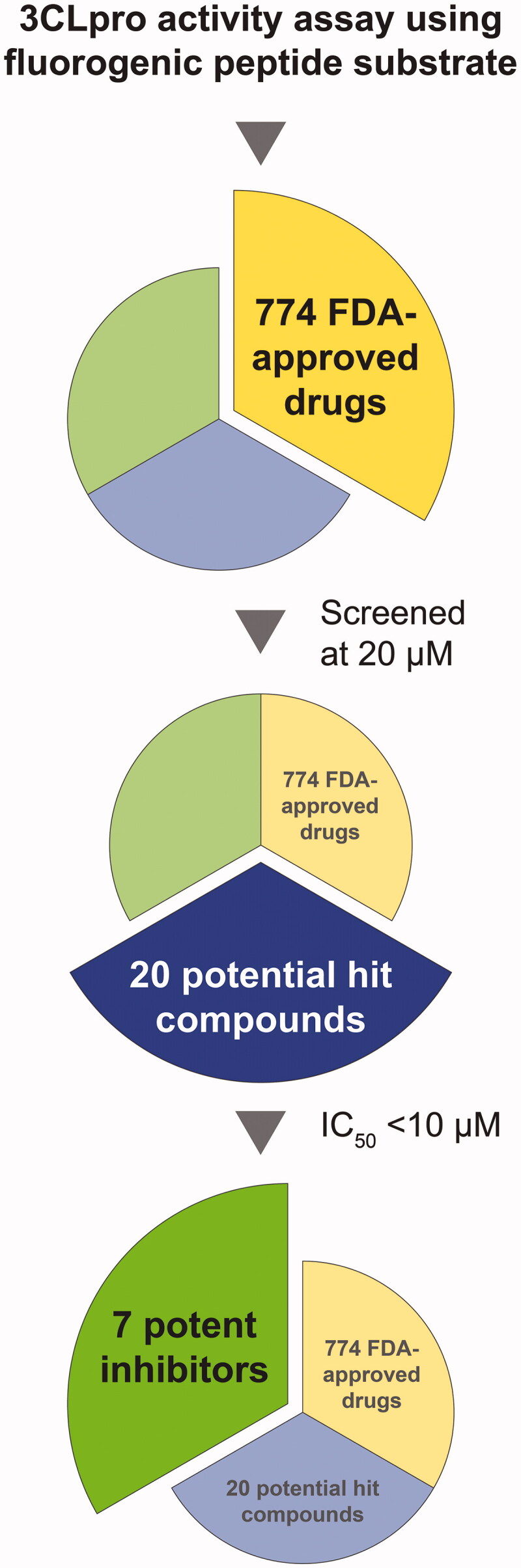
Flowchart of identification of SARS-CoV-2 3CLpro inhibitors in a library of 774 FDA-approved drugs. An initial screening was performed to evaluate the inhibition of SARS-CoV-2 3CLpro activity by FDA-approved drugs at 20 µM. Subsequently, IC_50_ characterisation was performed to pinpoint the more effective drugs. Twenty potential hit compounds were found, of which seven had a more pronounced effect in inhibiting SARS-CoV-2 3CLpro.

A compound library of FDA-approved drugs was screened for SARS-CoV-2 3CLpro inhibitory activity using an IQF peptide substrate. A flowchart of the screening procedure is shown in Figure 1. To identify compounds as potential SARS-CoV-2 3CLpro inhibitors, 774 FDA-approved drugs were screened at 20 µM in the high-throughput, initial screening. Among these 774 FDA-approved drugs, twenty potentially active compounds were found, including seven drugs with superior inhibitory activity against SARS-CoV-2 3CLpro. The twenty most active SARS-CoV-2 3CLpro inhibitors are listed in Table 1, with their IC_50_ values. Briefly, ethacrynic acid, naproxen, allopurinol, butenafine hydrochloride, raloxifene hydrochloride, tranylcypromine hydrochloride, and saquinavir mesylate led to 50% inhibition on SARS-CoV-2 3CLpro activity at concentrations below 10 µM. In addition, triptorelin acetate, goserelin acetate, rocuronium bromide, bisacodyl, armodafinil, and clobetasol propionate had an IC_50_ value of 10–20 µM, followed sequentially by seven moderate SARS-CoV-2 3CLpro inhibitors: sirolimus (rapamycin), colistin sulphate, cetirizine, bexarotene, cefpodoxime proxetil, clindamycin palmitate hydrochloride and oxaliplatin.

**Table 1.** Inhibition of SARS-CoV-2 3CLpro activity by FDA-approved drugs.

Compounds	IC_50_ (µM) <10 µM
Ethacrynic acid	1.11
Naproxen	3.45
Allopurinol	3.77
Butenafine hydrochloride	5.40
Raloxifene hydrochloride	5.61
Tranylcypromine hydrochloride	8.64
Saquinavir mesylate	9.92

Compounds	IC_50_ (µM) <50 µM
Triptorelin acetate	10.12
Goserelin acetate	12.02
Rocuronium bromide	17.47
Bisacodyl	17.51
Armodafinil	17.87
Clobetasol propionate	18.09
Sirolimus (Rapamycin)	22.30
Colistin sulphate	23.20
Cetirizine	25.58
Bexarotene	26.49
Cefpodoxime proxetil	32.43
Clindamycin palmitate hydrochloride	33.21
Oxaliplatin	47.13

### SARS-CoV-2 3CLpro inhibitors of therapeutic potentials

**Figure 2.**
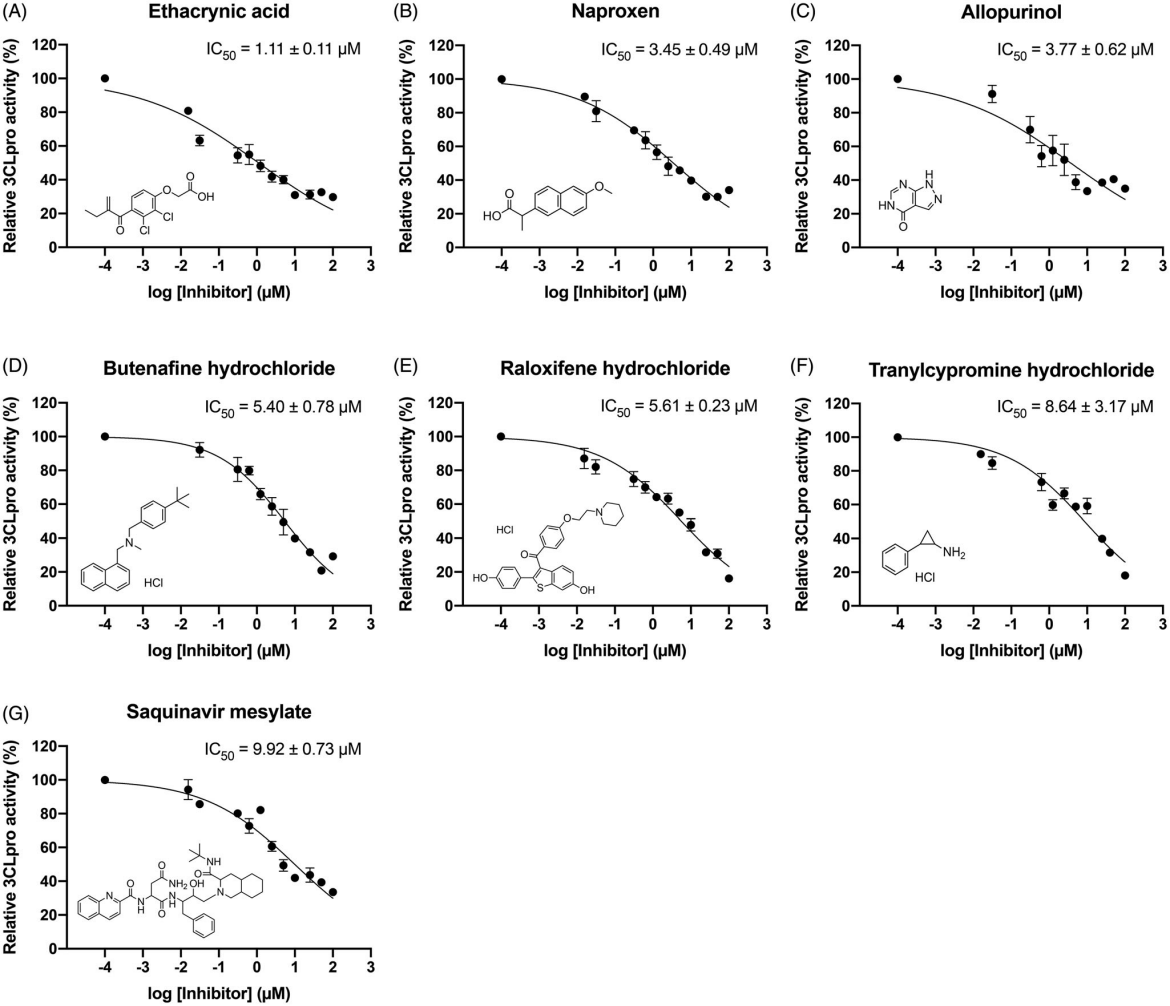
Dose-response curves of potent SARS-CoV-2 3CLpro inhibitors. The inhibitory activity of (A) Ethacrynic acid, (B) Naproxen, (C) Allopurinol, (D) Butenafine hydrochloride, (E) Raloxifene hydrochloride, (F) Tranylcypromine hydrochloride, and (G) Saquinavir mesylate against SARS-CoV-2 3CLpro are shown, along with a depiction of the chemical structure. Data (*N* = 3) are expressed as the mean ± SEM.

Regarding the therapeutic potential of the seven potent SARS-CoV-2 3CLpro inhibitors, dose–response curves of ethacrynic acid, naproxen, allopurinol, butenafine hydrochloride, raloxifene hydrochloride, tranylcypromine hydrochloride and saquinavir mesylate are shown in Figure 2, with their IC_50_ values and chemical structures. The measured IC_50_ values were 1.11 ± 0.11, 3.45 ± 0.49, 3.77 ± 0.62, 5.40 ± 0.78, 5.61 ± 0.23, 8.64 ± 3.17, and 9.92 ± 0.73 µM, respectively. Interestingly, despite the different protease family, saquinavir mesylate, an inhibitor of aspartate proteases^17^, was able to inhibit SARS-CoV-2 3CLpro, a cysteine protease.

### Molecular modelling of identified inhibitors in the substrate binding site of SARS-CoV-2 3CLpro

**Figure 3.**
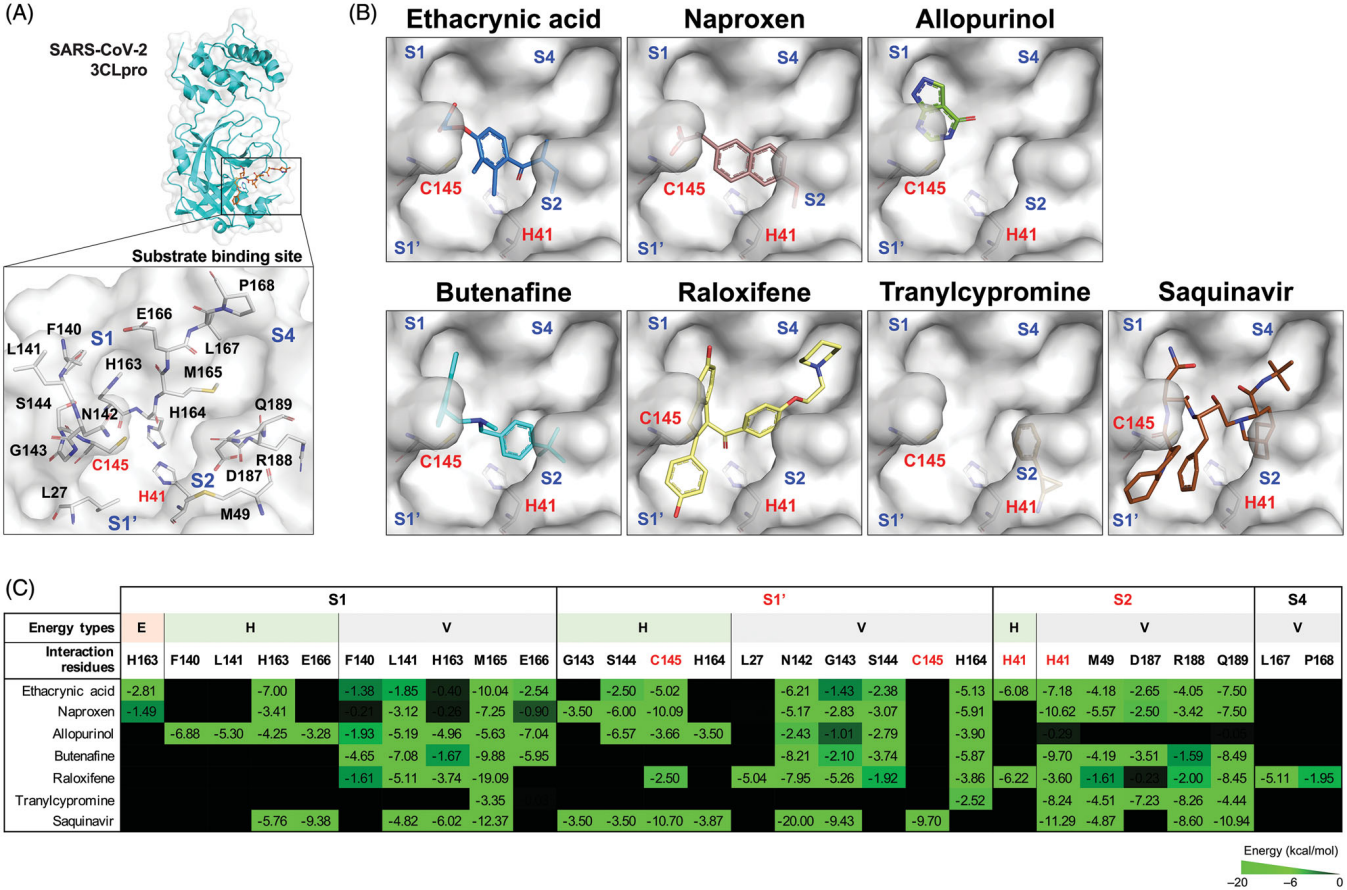
Interaction forces between the identified inhibitors and the substrate binding residues of SARS-CoV-2 3CLpro. (A) The substrate binding site of SARS-CoV-2 3CLpro. S1, S1’, S2 and S4 subsites are labelled in blue. Catalytic residues (red) H41 and C145, and other substrate binding residues (black) are labelled. (B) Molecular docking of seven SARS-CoV-2 3CLpro inhibitors. Substrate binding subsites (blue) and catalytic residues (red) H41 and C145 are labelled. (C) Interaction profiles of seven SARS-CoV-2 3CLpro inhibitors. The interaction energy (kcal/mol) positively correlates with the brightness of the colour (bright green). Catalytic residues H41 and C145 are labelled in red. E: electrostatic force (red fill); H: hydrogen binding force (green fill); V: van der Waals force (gray fill).

To elucidate the inhibitory mechanism of the identified SARS-CoV-2 3CLpro inhibitors, molecular docking was performed to simulate the binding model in the substrate binding site of SARS-CoV-2 3CLpro. As shown in Figure 3(A), the substrate binding site of SARS-CoV-2 3CLpro can be divided into four subsites^7^, where the S1 subsite comprises L27, N142, G143, S144, C145 and H164, the S1' subsite consists of H163, F140, L141, E166 and M165, the S2 subsite includes H41, M49, D187, R188 and Q189, and the S4 subsite is made up of L167 and P168. A way to disrupt the catalytic function of SARS-CoV-2 3CLpro is to occlude the access of the substrate to the Cys-His catalytic dyad (C145 and H41)^18^. The molecular docking results revealed that the identified inhibitors interacted with the Cys-His catalytic dyad, along with other residues, in the substrate binding site of SARS-CoV-2 3CLpro (Figure 3(B,C)). Specifically, ethacrynic acid and naproxen form a stable electrostatic force with H163 through the carboxyl group, and hydrogen bonding and van der Waals force with the catalytic dyad and other residues in the S1, S1' and S2 subsites. Butenafine interacted with the catalytic residue H41 and other residues in the S1, S1’ and S2 subsites through van der Waals force alone. Raloxifene and saquinavir filled all four subsites of SARS-CoV-2 3CLpro, binding to the catalytic residues C145 and H41, and the enclosed hydrophobic residues N142, G143, L141, M165, M49, L167, P168, R188 and Q189, resembling to the binding mode of Michael acceptor inhibitors^7^. As for compounds of a low heavy atom count, allopurinol and tranylcypromine occupied deeply in the S1 and the S2 subsite, respectively. Taken together, the identified inhibitors docked into up to four subsites of the substrate binding site of SARS-CoV-2 3CLpro, interacting with the catalytic dyad and other residues involving in substrate binding.

## Discussion

Coronaviruses, known for the crown-like appearance of the virions in electron microscopy, are enveloped + ssRNA viruses with the largest known genome size among RNA viruses. The genome encodes structural proteins (e.g. spike glycoproteins), non-structural proteins (e.g. papain-like protease (PLpro) and 3-chymotrypsin-like protease (3CLpro), helicase, RNA-dependent RNA polymerase), and accessory proteins^2^^,^^5^^,^^6^. SARS-CoV-2, a recently identified coronavirus, is responsible for the COVID-19 pandemic. In terms of societal demographics, the most vulnerable individuals are adults above 65 years of age, those with underlying conditions, and the economically disadvantaged^3^^,^^19^. Meanwhile, it has been determined that lymphopenia and elevated cytokine production resulting from SARS-CoV-2-induced immunopathology are responsible for disease progression and increased severity^20^. Based on the experience with SARS-CoV and MERS-CoV, active approaches to fight SARS-CoV-2 infection can be divided into three groups: (i) agents targeting the virus, (ii) agents targeting the host response, and (iii) spike-based vaccines^2^. Although the preliminary clinical data of vaccine development showed promise^19^^,^^21^, agents directly inhibiting viral replication remain of great interest. The current knowledge of β CoVs highlights the pivotal role of 3CLpro in viral replication and transcription and the value of developing broad-spectrum anti-β CoVs drugs in this regard^2^. Thus, 3CLpro inhibition has been regarded as a molecular approach in anti-SARS drug discovery and development^7^^,^^13^^,^^22^. Here, we screened a drug library consisting of 774 FDA-approved drugs for potential SARS-CoV-2 3CLpro inhibitors, using a protease-specific IQF peptide substrate.

Recently, treatment of severe COVID-19 patients with the HIV protease inhibitors lopinavir-ritonavir had no obvious efficacy beyond standard care^6^ but the final determination of their efficacy for COVID-19 patients requires further clinical study^23^. The use of hydroxychloroquine sulphate, an antimalarial agent, in severe or critically ill COVID-19 patients showed contradictory results in clinical trials^24^^,^^25^, and it is suggested to be more effective in early infection. Remdesivir, a nucleotide analogue prodrug in phase III clinical trials for Ebola virus infection, showed therapeutic promise for treating severe COVID-19 patients, with shortened recovery times^26–28^. Dexamethasone, a corticosteroid, was found to reduce the 28-day mortality of COVID-19 patients receiving either invasive mechanical ventilation or oxygen alone^29^. Based on the therapeutic experience against viruses, the most effective therapy for SARS-CoV-2 infection would most likely require a cocktail of agents targeting different stages of viral infection^30^. Indeed, combining lopinavir-ritonavir with two other agents helped alleviate symptoms, and a shortened viral shedding period was reported in mild-to-moderate COVID-19 patients^10^.

Utilisation of FDA-approved drug library is an effective and ideal tool for drug repurposing in antiviral research^7^^,^^31^, such as zika virus^32^, human rhinovirus^33^, and hepatitis B virus^34^. Regarding the possibility of using FDA-approved drugs for anti-SARS-CoV-2 therapy, we identified twenty potentially active drugs and these are listed in Table 1. Several of those drugs were previously reported to have antiviral activity. For example, ethacrynic acid derivatives have been shown to inhibit SARS-CoV 3CLpro activity by binding directly to the active site^35^. Naproxen was reported to be incorporated into the RNA-binding groove of the nucleoprotein of influenza A virus, suggesting its potential role in antiviral research^36^. The therapeutic potential of tranylcypromine for herpes simplex virus 1 (HSV-1) infection was evaluated because of its inhibitory activity against the histone-modifying enzyme, lysine-specific demethylase 1^37^. Raloxifene, a selective oestrogen receptor modulator, was reported to inhibit Ebola virus infection^38^. Saquinavir, the first HIV protease inhibitor made available in the market, was shown to be ineffective for inhibiting SARS-CoV replication^39^^,^^40^. Sirolimus blocked stages after the reverse transcription event in activated human T cells infected by human immunodeficiency virus 1 (HIV-1)^41^. Cetirizine, an antihistamine reported to inhibit the replication of respiratory syncytial virus (RSV) and the expression of interleukin-8 (IL-8), has an unknown property in reducing of RSV infectivity^42^. Bexarotene was shown to inhibit the expression of the hepatitis C virus core protein^43^. As for those that have not been mentioned, they have not yet been evaluated in antiviral research.

Importantly, a systematic review of the current evidence for non-steroidal anti-inflammatory drugs (NSAIDs) in the management of COVID-19 suggests that naproxen may be worthy of further investigation in clinical trials, because of its positive effects in controlling the symptoms of coryza, rhinovirus infection and influenza-related pneumonia^44^. On the other hand, the inhibitory activity of saquinavir against SARS-CoV-2 3CLpro denoted in this study matched the result from *in silico* molecular docking models reported previously^45^. Furthermore, sirolimus, a moderate SARS-CoV-2 3CLpro inhibitor identified in this study, was suggested to help prevent progression to severe forms of COVID-19 by mitigating the SARS-CoV-2-induced cytokine storm^30^^,^^46^. Last, but not least, bexarotene, a moderate SARS-CoV-2 3CLpro inhibitor, was shown to have broad-spectrum anticoronavirial activity in a study published recently^47^.

Taken together, we found several potent SARS-CoV-2 3CLpro inhibitors in a library of 774 FDA-approved drugs, including ethacrynic acid, naproxen, allopurinol, butenafine hydrochloride, raloxifene hydrochloride, tranylcypromine hydrochloride, and saquinavir mesylate. These drugs exert SARS-CoV-2 3CLpro inhibition by obscuring the accessibility of the C145-H41 catalytic dyad via hydrogen bonding and van der Waals force. Including the forces mentioned, the carboxyl group of ethacrynic acid and naproxen form an additional electrostatic force to H163 in the substrate binding site of SARS-CoV-2 3CLpro. Although ethacrynic acid had the best inhibitory activity against SARS-CoV-2 3CLpro, repurposing naproxen and sirolimus for COVID-19 treatment shows promise in that they have anti-inflammatory and immunosuppressive activities, respectively, which may help address the immunopathology induced by SARS-CoV-2 infection. Our identification of potent SARS-CoV-2 3CLpro inhibitors among FDA-approved drugs highlights their potential for treating COVID-19 and other diseases caused by β CoVs.
